# Failure to demonstrate short-cutting in a replication and extension of Tolman et al.’s spatial learning experiment with humans

**DOI:** 10.1371/journal.pone.0208794

**Published:** 2018-12-26

**Authors:** Stuart P. Wilson, Paul N. Wilson

**Affiliations:** 1 Department of Psychology, University of Sheffield, Sheffield, United Kingdom; 2 School of Life Sciences, University of Hull, Hull, United Kingdom; University of Lethbridge, CANADA

## Abstract

Successful demonstrations of novel short-cut taking by animals, including humans, are open to interpretation in terms of learning that is not necessarily spatial. A classic example is that of Tolman, Ritchie, and Kalish (1946) who allowed rats to repeat a sequence of turns through the corridors of a maze to locate a food reward. When the entrance to the corridors was subsequently blocked and alternative corridors were made available, rats successfully selected the corridor leading most directly to the food location. However, the presence of a distinctive light above the goal, in both the training and testing phases, means that approach to the light as a beacon could have been the source of successful short-cutting. We report a replication of the experimental design of Tolman et al. with human participants who explored geometrically equivalent virtual environments. An experimental group, who followed the original procedure in the absence of any distinctive cues proximal to the goal, did not select the corridor which led most directly to the goal. A control group, who experienced a light above the goal during training and testing, were more likely to select a corridor which led in the direction of the goal. A second control group experienced the light above the goal during training, but in the test the location of this cue was shifted by 90° with respect to the start point of exploration. This latter group responded unsystematically in the test, neither selecting a corridor leading to the original goal location, nor one leading directly to the relocated light cue. The results do not support the hypothesis that travelling a multi-path route automatically leads to an integrated cognitive representation of that route. The data offer support for the importance of local cues which can serve as beacons to indicate the location of a goal.

## Introduction

Is spatial learning distinct from other forms of learning? According to associative learning theories, when we encounter new information, whether temporal or spatial, that information is processed according to the same associative mechanisms ([1], see also [2–5]). In contrast, according to cognitive mapping theory [6, 7], when we explore a new environment we automatically construct a representation of that environment in which all distinctive landmarks, together with the geometric relationships between those landmarks, are stored. A mental representation of the environment in this structure can be employed to solve novel spatial problems. As the geometric relationships in a cognitive map are vectorial they do not conform to the accepted description of Humean associations, which comprise links between ideas or sensations [8]. Therefore, when vectorial knowledge is assumed to underpin navigation, the mechanisms that underpin spatial and associative learning are presumed to differ.

There are many ways to reach a goal. In their classic discussion of spatial learning and navigation O’Keefe and Nadel (1978) [7] distinguished between taxon and locale systems. They suggest that the taxon system incorporates ‘orientation’ and ‘guidance’ mechanisms, which essentially refer to stimulus and response associative learning. The locale system, by contrast, processes true spatial knowledge which forms the basis of an allocentric cognitive map. While navigation might rely on either system depending on the cues that are available, cognitive mapping and associative learning theories make a number of different predictions about the way in which humans and other animals process spatial information. For example, associative learning theories predict cue-interaction effects, such as the competition between spatial cues found in a blocking design [9–11]. In this experimental arrangement, stimuli which have already been established as good predictors of a goal location, can restrict what interrelationships are learned about newly introduced stimuli and that goal [12, 13]. In contrast, cognitive mapping theory maintains that an internal representation of spatial interrelationships is only useful if it is automatically updated when changes occur in the environment. Therefore, under circumstances in which cognitive mapping forms the basis of navigation, phenomena such as blocking should not occur.

One important prediction which differs between associative learning theories and cognitive mapping theory is that only cognitive mapping predicts that we can compute a short-cut based on geometric interrelationships. For example, to learn a route by exploring one path from landmark *a* to landmark *b*, and another from landmark *a* to landmark *c*, and then be able to select a new short-cut from landmark *b* directly to landmark *c*.

**Fig 1:**
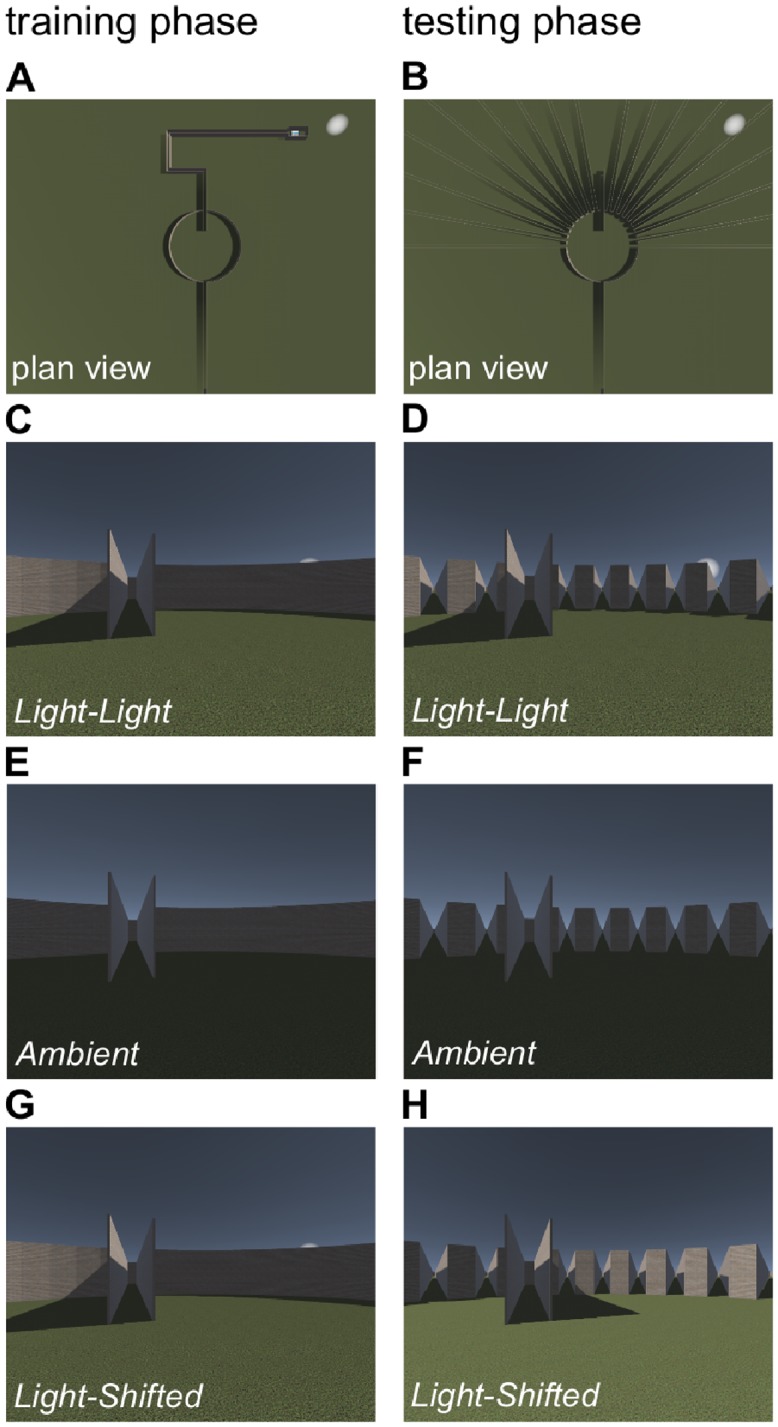
A replication of the experiment of Tolman, Ritchie, and Kalish (1946) in a virtual environment. **A** Plan view of the layout of the environment in the training phase. Participants begin at the Southernmost point and negotiate a sequence of turns through narrow corridors to a goal located in a square chamber to the North-East. A distinctive light source (for groups *Light-light* and *Light-Shifted*) is located above and adjacent to the goal. **B** Plan view of the testing environment, in which the original route to the North is blocked and eighteen additional routes are made available. **C** First-person view from the center of the circular chamber in the training environment in the *Light-light* condition. **D** First-person view from the center of the testing environment in the *Light-light* condition. **E-F** First-person views from equivalent locations in the training and testing phases in the *Ambient* condition. **G-H** First-person views from equivalent locations in the training and testing phases in the *Light-Shifted* condition. Note that panels **C** and **G** are identical.

Early evidence in support of the prediction of short-cutting from cognitive mapping theory is provided by the seminal study by Tolman, Ritchie, and Kalish (1946) [14] (henceforth referred to as Tolman et al.), who gave rats repeated experience of navigating a fixed route through a maze comprising several turns, before presenting them with an altered version of the environment in which multiple alternative routes were available (see also [15]). The training phase consisted of a straight path into a circular chamber, leading directly ahead to an entrance corridor, followed by 90° turns to the left, then right, then right again, to proceed down a longer path than the others to a food goal (see Fig 1A); this route was repeated over several days. The testing phase involved a single trial in an altered version of the environment in which the original path to the food was blocked, and 18 alternative pathways (at 10° increments) were made available (see Fig 1B). Tolman et al. reasoned that in this scenario a cognitive map would lead the animals to enter the corridor that faces directly toward the location in which the reward was experienced during the training phase (toward the East of the starting location, assuming that the first direction of travel during training is considered North, i.e. upward on Fig 1A and 1B). Alternatively, associative learning theory would predict that animals would have originally learned to find the goal by approaching the entrance corridor, then moving forward, making turns as required to reach the food. This latter strategy does not involve knowledge which can be used to deduce a short-cut to the goal. In the test phase, predictions from this account include selecting a pathway immediately adjacent to the originally trained corridor due to generalisation [16, 17]. Conceivably, selecting a pathway to the West is a plausible alternative if generalizing from the first turn, to the left, during each training trial was sufficient to elicit a positive association with the food [5] (see also [18]).

In the test phase of the original study, the rats overwhelmingly chose to explore a route pointing directly to where the reward had previously been experienced (to the overall East of the orientation of the blocked corridor). This result has been cited as evidence for the ability of animals to compute short-cuts, and thus as evidence in support of cognitive mapping theory. However, the original experiment has a well known potential flaw in its design [4, 19]. In the original study a lightbulb was present above and adjacent to the goal, and was present in both the training and the testing environments. Therefore, it is possible that choosing the path that led to the goal in the testing phase did not reflect the construction of a cognitive map of the route by integrating spatial information about the distances and angles between spatial features in the training phase.

At least two alternative accounts of the original results of Tolman et al. are plausible. The most parsimonious explanation is that in the absence of any learning during the training phase, the animals may have navigated toward the light as the most distinctive feature of the test environment. Alternatively, the animals may have learned during the training phase to associate the approach to the light with the subsequent delivery of a food reward. This latter account is consistent with a somewhat weaker interpretation of the original data offered by Tolman et al.; “…we believe that it is not correct to say that our rats were merely running towards the light. Rather we should say that they were running to the position of the former goal, and that this location was indicated by the position of the light” [14].

Indeed, a number of reviewers have argued that there are no unequivocal demonstrations of successful short-cutting in the absence of the opportunity for guidance by landmarks proximal to the goal [2, 3]. For example, studies with a range of species suggest that when a distinctive landmark consistently appears at a short distance from a rewarded location, the landmark may come to serve as a ‘beacon’, and as such elicit a behavioural strategy in which the animal first approaches the beacon and then searches locally for the goal, either with respect to additional directional information [20, 21] or by randomly searching outwards from the beacon. Before assuming a more complex process, such as the integration of spatial information to form a route between start location and goal, we need to know more about the role of local cues in short-cutting. As Mackintosh [3] notes: to the extent that there are many ways by which animals can find their way directly to their goal, they may have little need to develop an integrated map of an entire environment. While there is evidence that animals can successfully take a short-cut based on path integration or dead reckoning [22], we know of no unequivocal demonstrations of short-cutting that are necessarily based on the integration of cognitively based route knowledge. The absence of evidence for successful short-cutting is not the same as evidence for an inability to take short-cuts, hence by appropriately modifying the original experimental design of Tolman et al., we will here seek evidence for short-cutting.

Despite the potential shortcoming in Tolman et al.’s experiment, the basic methodology can readily be extended to provide a sound test of the hypothesis that an integrated cognitive spatial structure underpins short-cutting. The present study was designed to investigate short-cutting in people exploring a virtual environment in a design which replicated the essential features of the original experiment of Tolman, Ritchie, and Kalish (1946) [14] with rats (see Fig 1 for an overview of the design). To address the potential criticism related to the light in the original experiment, one group of participants, group ‘Ambient’, was trained and tested in the simulated environment under conditions of unlocalised ambient lighting. The question of interest for this group was whether, in the absence of the light-beacon, they would make similar choices to those of Tolman et al.’s rats and select test-paths that were oriented in the direction of the goal. This outcome would be entirely consistent with cognitive mapping theory, at least in the human case. Or, whether they would choose paths adjacent to the path that was available during the training phase as a ‘generalization’ account might predict. A second group, group ‘Light-Light’, replicated Tolman et al.’s procedure in having a light above and adjacent to the goal in both the training and test phases. The question of interest for this group was whether more participants would select a path that was oriented toward the light than in group *Ambient*. Should significantly more people choose a path directed toward the light than in group *Ambient*, this would reinforce the conclusion that the light acts as a beacon that indicates the goal location. To the extent that short-cutting is based on a cognitive map, a significant difference between groups *Ambient* and *Light-Light* would not be expected. Finally, group ‘Light-Shifted’ replicated Tolman et al.’s procedure in having a light above and adjacent to the goal in the training phase, but differed in that during the test the light was moved to a different location within the environment. Should path selection in the test be based purely on the beacon properties of the light, participants should select the path that leads toward the relocated light. Should a conjunction of the light and the spatial features of the environment guide the choice of path, selection would be more varied. Given that the light location in the test differs from that during training, in the test the light cannot support approach to the original light location in this group. Therefore, in group *Light-Shifted* fewer arm choices consistent with the learning phase would be anticipated due to generalization decrement.

## Methods

### Overview

The experimental procedure was designed to reproduce the essential features of the study reported by Tolman, Ritchie and Kalish (1946) [14], using human participants instead of rats as subjects. The essential geometrical features of the original training and testing arenas were recreated using 3D modelling software to create a series of virtual environments (VEs) with geometries that corresponded precisely to the training and testing environments used by Tolman et al. Participants were immersed in VEs using a head-mounted display, and they navigated through the VEs by pointing a handheld controller. Participants were instructed to search for a goal location, which was marked by a distinctive image. All participants completed seven trials in the training phase, navigating the same route through narrow corridors to the goal location, followed by a single trial in which the original route was blocked and 18 alternative routes were made available (see Fig 1A and 1B). Participants then returned to the training environment and were asked to point in the direction of the goal location. Finally, participants were asked to draw a plan view of the training environment on a sheet of A5 paper.

### Design

A between-participants experimental design was employed to investigate the possible influence of the light in the training and/or testing phases on participants choice of path during the testing phase. Three experimental conditions were defined by the location and presence/absence of a distinctive light source in the training and test versions of the VE. In the *Ambient* condition, no distinctive light source was present in either the training or the test phase, and the environments were instead illuminated by ambient lighting that was not localised and did not cast shadows. Beyond making the walls and floor of the environments visible the ambient light provided no additional information about the geometry of the training or test environments. In the *Light-Light* condition, a distinctive circular light source was present above and adjacent to the location of the goal in both the training and the testing phases (i.e., North-East of a central circular chamber), recreating the conditions tested with rats by Tolman, Ritchie, and Kalish (1946) [14], in which the presence of the landmark in the testing phase provided additional information about the location of the goal. In the *Light-Shifted* condition the distinctive light was present as for group *Light-Light* in the training phase, and then moved to a different location during the testing phase (North-West of the central chamber), thus any association formed between the light and the goal during the training phase would make the presence of the light in the testing phase a source of direct conflict with any learned spatial representation. Dependent variables were choice of path in the test, goal-direction pointing accuracy, and the assessed quality of plan views of the training VE drawn by participants on completion of the test phase.

## Participants

Participants were thirty males and thirty females distributed equally across three conditions (ten males and ten females in each condition), with a mean age of 20.33±0.97 years (mean ± standard deviation). The study received ethical approval from the ethics committee of the Department of Psychology at the University of Sheffield. Participants provided informed consent to participate in ‘a study on spatial learning in virtual environments’, were informed about the procedure, and were fully debriefed about the scientific aims of the experiment following data collection.

### Materials

Virtual environments (VEs) were created and rendered using the freely available software Unity 3D [23], which provides an interface to several commercially available virtual reality hardware devices, and allows custom scripts to be written in the c-sharp programming language. Participants were immersed in the VEs using a HTC Vive head-mounted display, the location and orientation of which was tracked by two externally mounted sensors positioned to define a tracking volume in which participants could safely take two or three strides away from the origin. The headset was tethered to a personal computer by a 5 meter cable held by an assistant experimenter to safeguard against tripping. The location and orientation of the participant in the VE could be changed by physically moving, but prolonged translations at a pre-specified fast walking pace, equivalent to 2.2 meters per second (or 5 miles per hour), were achieved by pointing a handheld controller and holding down the top or bottom of a button directly underneath the thumb to walk forwards or backwards respectively. Based on pilot experiments carried out during software development, this control strategy was found to be the most intuitive for participants to adopt, because it allowed the walking direction and the head-direction to be decoupled, and because a variety of strategies could be used to change the walking direction, e.g., by rotating the wrist, the arm, the entire trunk, or by stepping. Between trials, participants were transported instantaneously by a key-press from the experimenter. The experiment was run using a purpose-built personal computer, which included an NVIDIA GeForce GTX 970 graphics processor, for low-latency rendering of the VE graphics and communication with the virtual reality hardware.

### Procedure

#### Pre-training

In the initial ‘pre-training’ phase, participants were required to spend at least one minute exploring a practice VE, adjusting to wearing the virtual reality headset, practicing with the use of the handheld controller to walk around, and communicating with the experimenter to report any feelings of discomfort or raise any queries. The environment consisted of a large circular chamber, with uniform brickwork textures mapped on vertically oriented polygons to indicate the presence of walls, and a uniform grass texture mapped onto the ground plane to indicate a floor. The radius of the circle corresponded to 20 meters, and the height of the virtual walls corresponded to 4.36 meters. The pre-training environment was illuminated by non-directional ambient lighting. On the floor 15 meters from the center of the chamber was a large dark square box (4 by 4 by 1 meters) and onto its top face was rendered the image of a packet of potato chips. Participants were encouraged to explore the box, and instructed that when the experiment began they would be asked to ‘find the crisp packet’.

#### Training phase

In the second phase, referred to as the ‘training phase’, participants were transported instantaneously to the start location to the ‘South’ of the training VE, at the end of a narrow corridor connected to the original circular chamber. The box displaying the crisp image was moved to a goal location which could be reached by walking directly across the circular chamber from the start location, entering a short corridor, and taking 90° turns to the left, then right along short corridors, then right again to a final longer corridor that opened out into a small square chamber housing the box that contained the crisp packet image. In the *Light-Light* and *Light-Shifted* conditions a light source was visible as a large bright circular object 18 meters behind the location of the goal (from the angle of approach along the final corridor) and raised to 10 meters from the ground plane, above the height of the walls. The light was oriented to the West and angled at −45° relative to the ground plane, i.e., pointing towards the floor and down the corridor from which the goal was approached, recreating the conditions described by Tolman et al. [14]. When the participant reached the goal location and the goal was clearly visible, the experimenter asked the participant ‘Are you ready for another go?’, and when the participant confirmed, the experimenter pressed a key to instantaneously return the participant to the beginning of the training route. This procedure was repeated on a total of seven occasions.

#### Testing phase

At the end of the seventh trial in the training environment, participants were again asked ‘Are you ready for another go?’, and when the participant confirmed, the experimenter pressed a key to instantaneously move the participant to the beginning of an altered testing arena. The participant’s view had the same relative location and orientation as during training. In the testing environment the original pathway led to the central circular chamber as before, but the original pathway to the goal was blocked just before the first 90° turn that had been available on training trials. Eighteen additional corridors were available at 10° increments around the Northern half of the central circular chamber (see Fig 1B). In the *Light-Light* condition the light was in the same location (69 meters East and 63.5 meters North of the center of the circular chamber), and oriented to the West, as it was in the training environment. In the *Light-Shifted* condition the light was moved to the opposite location about the North-South axis (69 meters West and 63.5 meters North of the center of the circular chamber), and oriented to the East, again angled at −45° relative to the ground plane. If necessary, the instruction to ‘find the crisp packet’ was repeated. When participants had moved at least one third of the way down one of the newly available corridors they were instructed to stop and their choice was recorded. This procedure (experience of the testing arena) was completed once only.

#### Goal orientation test

Participants were again asked ‘Are you ready for another go?’, and upon confirmation the experimenter pressed a key to instantaneously move the participant to the beginning of the environment that they had explored in the training phase. Participants we instructed to walk to the center of the circular chamber (recreating the conditions of the initial approach in the previous phases), and when approximately at the center they were asked to stop walking, and to ‘Point the handheld controller in the direction where you think the crisp packet is …pull the trigger on the back of the controller so that the computer can record your decision’. The computer recorded both the location and the orientation of the handheld controller at the pull of the trigger, such that measures of pointing accuracy could appropriately account for variations in the location of the participants at the time of pointing.

On completing VE exploration, participants were given approximately 30 seconds to adjust once the headset was removed, seated at a desk with a sheet of A5 paper and a pencil, and asked ‘Please can you try to sketch the layout of the environment that you were just in. Try to be as accurate as possible.’ Finally, participants were thanked for their participation and the scientific aims of the experiment were explained. Drawings were subsequently labelled with a unique identification number and electronic scans of all drawings were provided for blind rating.

## Results

**Fig 2:**
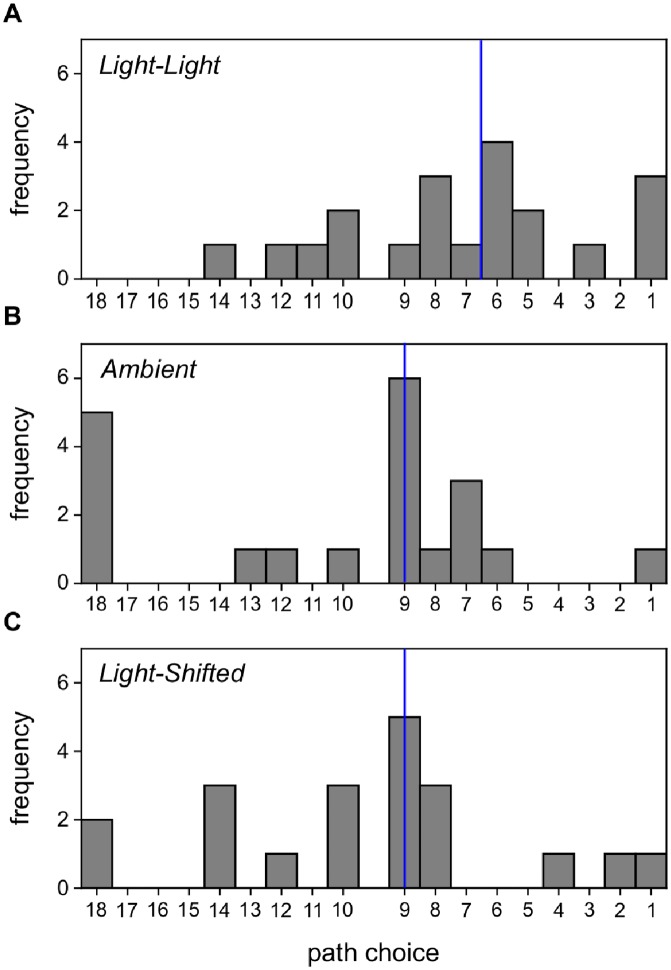
Histograms showing the distribution of paths chosen in the testing phase of the three groups. **A** Path choices in the *Light-Light* condition. **B** Path choices in the *Ambient* condition. **C** Path choices in the *Light-Shifted* condition. Paths are labelled in reverse order, such that paths in Westerly directions appear towards the left and paths in the Easterly directions appear towards the right. The path between choices 9 and 10 was blocked in the testing phase. Path 6 corresponds to the path leading directly to the location at which the target was located in the training phase. Blue vertical lines indicate the median for each distribution.

To analyse the distribution of path choices, the 18 possible paths that participants could choose in the test phase were labeled in terms of the anti-clockwise ordering of the angles at which they projected from the center of the large circular chamber, starting from path 1 at 0° (East) and incrementing to path 18 at 180°. As the original path to the North was blocked and some paths were not selected, while some were selected by relatively few participants, non-parametric analyses were employed to compare the median numbers of the paths chosen between groups. The median angle of the paths chosen by participants in the *Ambient* and *Light-Shifted* conditions was 80° (path 9). The median angle of the paths chosen by participants in the *Light-Light* condition was 55° (between paths 6 and 7). A Kruskal-Wallis test on the path choices revealed a significant difference between the median scores; *H* = 7.89, *p* = 0.02. To explore the significant difference between groups, Mann-Whitney U pairwise tests were carried out for the three pairs of conditions using a Bonferroni correction. The median path chosen in the *Light-Light* condition was significantly different from the median path chosen in the *Ambient* condition (*U* = 106, *p* = 0.01, two-tailed test). The difference between the median path chosen in the *Light-Light* condition and in the *Light-Shifted* condition approached but did not meet the corrected significance level (*U* = 116.5, *p* = 0.023, two-tailed test) (Bonferroni correction, *p* = 0.017). No difference was found between the median path choices in the *Ambient* versus *Light-Shifted* conditions (*p* > 0.05). Histograms of the chosen paths are presented in Fig 2.

These analyses suggest that participants in group *Ambient* tended to choose paths adjacent to the path that was available during the training phase, as a ‘generalization’ account might predict. They did not make similar choices to those of Tolman et al.’s rats, and did not conform to Tolman’s expectation based on a cognitive representation of the route. In the *Light-Light* condition, a replication of the condition tested by Tolman, Ritchie, and Kalish (1946) [14], participants were more likely to select a path oriented toward the goal/light location than in the *Ambient* condition in which no distinctive landmark provided spatial information about the location of the target. This outcome conforms to the expectations of Tolman et al. based on short-cutting, and reinforces the conclusion that the light can act as a beacon that indicates the goal location in this procedure. In group *Light-Shifted*, path selection was not purely based on the beacon properties of the light. If it had been, in the test participants should have principally selected the path leading toward the new location of the light. In this group the (weak) tendency was to approach the paths adjacent to the originally trained path, suggesting that movement of the light location from that during the training phase disrupted learning that the goal was in a North-Easterly direction: a generalization decrement. Nonetheless, the difference in path choices between groups *Light-Light* and *Light-Shifted* reinforces the importance of the light as a landmark.

**Fig 3:**
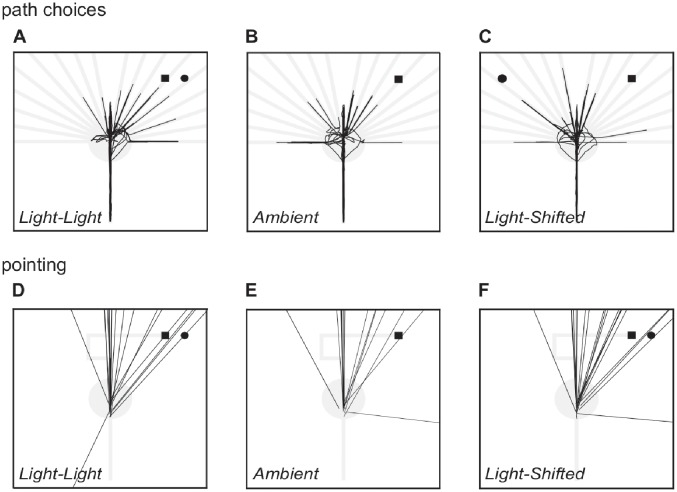
Movement patterns in the testing phase and participant estimates of the direction toward the goal in the goal orientation test. **A-C** Movement patterns for each of the 20 participants in each condition are shown overlaid as black traces. The light grey image shows the layout of the environments in the testing phase. A black square indicates the position of the goal, and a black circle indicates the position of the light. **D-F** Pointing directions for each of the 20 participants in each condition are shown as overlaid black lines, originating from the location of the handheld controller at the time of pointing, and extending in the direction at which the controller was oriented.

Pointing accuracy was defined as the absolute difference, in degrees, between the actual direction to the goal location and the participant’s estimate of that direction. Groups did not differ significantly in accuracy, with mean absolute errors for groups *Ambient* = 32.4°, *Light-Light* = 33.4°, and *Light-Shifted* = 26.3°, (*p* > 0.05). Fig 3 shows the direction in which each participant pointed for each condition. Absolute pointing errors were subject to a between-participant ANOVA analysis with group and gender as factors; this analysis revealed no main effects and no interaction between main effects (ps > 0.05).

Inspection of the individual trajectories (see Fig 3A–3C) revealed that approximately half of the participants across the groups explored the blocked path prior to making their recorded path choice (50% in condition *Light-Light*, 65% in condition *Ambient* and 50% in condition *Light-Shifted*), and that the vast majority of these approached the center of the circular chamber before directly approaching and exploring only the path that was subsequently recorded.

**Fig 4:**
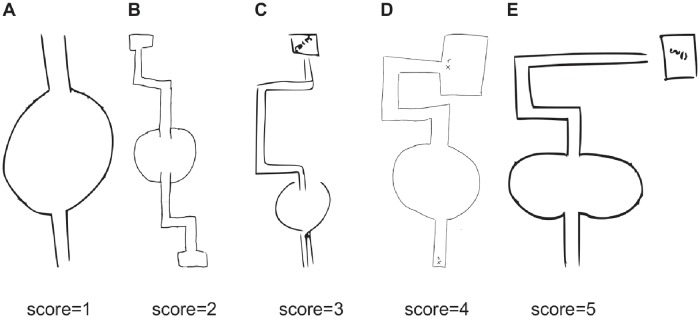
Examples of drawings of the layout of the virtual environment in the training phase. Drawings are arranged from left to right to reflect increasingly good scores assigned by a blind rater. **A** Drawing by a female participant assigned to group *Light-Light*, scoring 1/5. **B** Drawing by a male participant assigned to group *Ambient*, scoring 2/5. **C** Drawing by a female participant assigned to group *Light-Shifted*, scoring 3/5. **D** Drawing by a male participant assigned to group *Ambient*, scoring 4/5. **E** Drawing by a male participant assigned to group *Light-Light*, scoring 5/5.

The plan views of the training environment, drawn after completion of the test procedure, were blind-rated according to an ordinal 5-point scale from ‘*1. Inaccurate plan which is not reminiscent of the actual space*’ to ‘*5. Accurate plan view with 4 paths leading from the centre, in the correct arrangement. Angle from centre of circle to the ‘goal’ within 10° either way’*. An example drawing for each of the five ratings is shown in Fig 4. The median rating for all three groups was 4 (‘*Accurate plan view with 4 paths leading from the centre, in the correct arrangement. Angle from centre of circle to the ‘end’ greater than 10° either way, but less than 90°*’). The majority of drawings were rated 4 (62%) with the remainder receiving ratings of 5 (10%), 3 (12%), 2 (14%) and 1 (2%). No significant difference was found between group medians, Kruskal-Wallis *p* = 0.13. Most participants drew fairly accurate plan views with the most common deviation, for those receiving scores of 4, a tendency to make the final path to the goal of similar length to the other paths leading from the central circular chamber. This tendency resulted in plots of the goal location that were shifted toward the West of the actual location, conforming to the majority of path choices across groups. While this finding is suggestive that map-like representations might have guided path choices, more detailed individual comparisons do not conform to this view: Correlations between map ratings and path choices were near zero for participants collectively across groups, Spearman’s rho *ρ* = −0.14, *p* > 0.05, and for each group individually, Spearman’s rho *ρ* < 0.1, *p*s > 0.05. Similarly when accuracy of pointing toward the goal (in degrees) was compared with map ratings (ordinal scale 1-5), correlations were near zero for participants collectively across groups, Spearman’s rho *ρ* = −0.14, *p*s > 0.05, and for each group individually, Spearman’s rho *ρ* < 0.1, *p*s > 0.05. Thus, map drawing quality and wayfinding measures were unrelated, which would not be expected if path-choice and goal-pointing relied on the same knowledge as that used to draw the maps.

While there was no gender effect for any individual group (*p*s > 0.05), there was an overall (all groups combined) difference in map ratings with mean map ratings of 3.9 for men (range 2-5) being higher than the mean map ratings of 3.6 for women (range 1-5), Mann-Whitney U, *p* = 0.04. Separate analyses of path choice and pointing error data for those participants whose map drawings were rated 4 or 5 (group *Light-Light* *n* = 12 participants, group *Ambient* *n* = 12, group *Light-Shifted* *n* = 18) presented a very similar statistical picture to analyses of all participants’ data as reported above.

## Discussion

This study replicated with human participants the essential features of the seminal short-cutting experiment by Tolman, Ritchie, and Kalish (1946) [14] with rats. In Tolman et al.’s study a distinctive light above the location of a food reward was present when the animals were trained and tested. As Tolman et al. noted (but rejected by argument), this cue could have acted as a beacon that guided path choice in the test. Therefore, we included groups to evaluate the influence of the light in addition to a group, group *Ambient*, which assessed short-cutting free from the influence of this potential confound.

Replication of the original experimental design in the *Light-Light* condition using humans in virtual environments rather than rats as subjects, yielded similar short-cutting behaviour in humans, with the median and modal path choice in the testing phase both pointing in the direction that was rewarded during training. This pattern is consistent with similar spatial strategies in rats and humans.

In the absence of the beacon, participants in group *Ambient* tended to make path choices in the test that were adjacent to the location of the path that led away from the central chamber during the training phase. This pattern does not conform to that anticipated by Tolman et al., which assumes path choices in the test that were based on the shortest route to the goal location experienced during training. However, in group *Light-Light*, a close replication of the arrangement in the study of Tolman et al., path choices tended toward the paths leading to the goal/light. This pattern is what would be expected if, contrary to their arguments, the light acted as a beacon indicating proximity to the goal. That participants chose paths in the direction of the goal (path 6) more often than paths in the direction of the light (path 5) suggests that the availability of the light as a cue for learning during the training phase allowed it to be used to localize the goal during testing, and suggests that participants were not simply approaching the light in the test phase. In group *Light-Shifted*, path choices were centred around path 9, as they were in group *Ambient*, rather than tending toward path 14 as would be expected purely on the basis of the light’s properties as a beacon. This pattern suggests that path choices were determined by more than the beacon properties of the light. Moving the light location and reversing its facing direction, with concomitant changes to the direction of the shadows, appears to have engendered a generalization decrement in the goal-location information within the spatial array as a whole. Overall, these findings are consistent with those obtained using related experimental designs with humans in virtual environments, demonstrating a lack of accurate short-cutting behaviour in the absence of salient landmarks, an improvement in short-cutting when stable landmarks are present, and a disruption of short-cutting when landmarks are shifted between training and testing phases [24, 25].

In the present study we tested participants in virtual environments for their obvious practical advantages, and because there is good evidence that findings from exploration translate and transfer between VE and real-world scenarios [26, 27]. Of course, a VE can never capture every aspect of the original Tolman et al. study. It is conceivable that path choices in rats were influenced by additional spatial cues that were presented in a consistent relationship with the reward location in both training and testing phases, e.g., extra-maze visual landmarks (doors, ceiling corners, equipment etc.), smells or sounds. However, the present data collected in the *Light-Light* condition by exploration of precisely controlled VEs confirm that in combination with the geometry of the training and test arenas, the presence of a single consistent distinctive landmark alone is sufficient to guide path choices to a previously rewarded location.

Unlike the rats of Tolman et al., our participants were able to supply direct evidence of map-like knowledge when asked to draw the layout or plan view of the training environment. In the median-rated case for each group, these plan views were generally reflective of the space explored during training, in that the general arrangements of spatial features was quite accurate. A frequently observed limitation on accuracy was a tendency to draw the final path to the goal as shorter than its actual relative length; rather, this path was depicted as of a similar length to the other paths (see Fig 4D). It is possible that the shortening of the final drawn path reflects a mismatch in the available proprioceptive feedback, which in our setup (with a head tracking volume comprising a few cubic meters) was informative about rotations of the head and body, but less informative about distance.

The generally high accuracy of participants’ drawings is nonetheless consistent with the possibility that path selection was based on an internal representation with map-like qualities. However there are several reasons for questioning this assumption. First, the accuracy of the plan-view drawings was very similar for all groups; therefore, had map-like knowledge formed the basis of path selection, we would not expect a difference in median path choices between groups. That there was a difference between median choices suggests a different selection mechanism between groups. Second, the accuracy of the plan-views seems at odds with the relatively disparate path choices within each group (see Fig 2). Indeed, the rats of Tolman et al. presented a more consistent pattern of path choices than our participants, with 36% of rats choosing path 6, compared with only 20% of human participants in group *Light-Light*. Surely there would be greater consistency in path choices if these were guided by maps of similar structure. With respect to the apparently low consistency of human choices compared with rats, note that the rats in Tolman, Ritchie, and Kalish (1946) were reportedly ‘maze-wise’, having concluded 18 days of maze training immediately before the experiment (see also [16, 28]). Such training should facilitate the short-cut task by habituating the rats to the general maze-learning environment and procedure.

Perhaps the most important reason to question a plan-view basis for short-cut performance is the essentially zero correlation between map accuracy ratings and the accuracy of pointing toward the goal, and a similar lack of correlation between map accuracy ratings and path choices. That is, the significant differences that we found between groups were manifest at the time at which any such map should first have been invoked to guide navigation behaviour. Given these observations, we suggest that plan views were likely deduced when requested, subsequent to the operation of mechanisms which actually determined pointing accuracy and path choice. The main mechanism leading to a short-cut choice in this procedure appears to be using the light as a beacon to locate the goal (cf. [3]).

A wealth of important data is accumulating related to patterns of neural activity during spatial exploration (see [6, 29] for reviews), with many researchers concentrating on the hippocampal formation as the underlying substrate for the construction and maintenance of cognitive maps. However, there remains a surprising lack of behavioural evidence to support the idea that humans or other animals routinely maintain veridical representations of spatial interrelationships which can be used to solve problems such as short-cutting. Our current data broadly reinforce this conclusion, with the only evidence for successful short-cut performance found in a group who were given the opportunity to use information which does not reflect truly spatial knowledge: approach to a beacon. Therefore, we await more conclusive evidence to implicate truly spatial knowledge in spatial problem solving. Meanwhile we maintain conservative assumptions about the ability of people to derive novel routes from stored representations of spatial interrelationships.

## Supporting information

S1 Data Anonymised data record. (CSV)
